# Chemical synthesis-based structure correction of a claimed protein-free antifreeze oligosaccharide

**DOI:** 10.1093/nsr/nwae364

**Published:** 2024-10-17

**Authors:** David Crich

**Affiliations:** Departments of Pharmaceutical and Biomedical Science, and Chemistry, and Complex Carbohydrate Research Center, University of Georgia, USA

**Figure 1.**
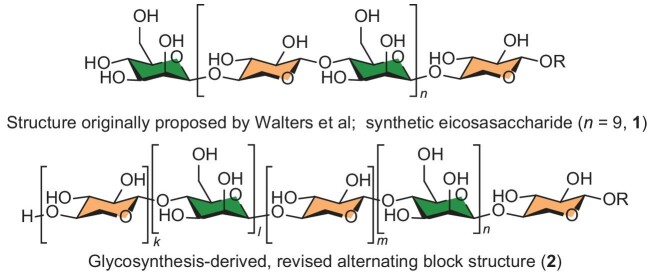
Structures of the initially proposed alternating xylomannan and of the synthetic eicosasaccharide (**1**) lacking TH properties, and of the revised alternating block structure (**2**) consistent with the NMR data but still lacking TH properties.

Psychrophilic organisms living at sub-zero temperatures produce antifreeze proteins (AFPs) or antifreeze glycoproteins (AFGPs) to prevent the growth of cell-membrane- and organelle-damaging ice crystals. AFPs and AFGPs function by binding to ice crystals and in doing so change their morphology and induce thermal hysteresis (TH), the lowering of the freezing point below the melting point, thereby preventing crystal growth. A 2009 report of an antifreeze xylomannan with an average molecular weight of 2.9 kDa from the Alaskan freeze-tolerant beetle *Upis ceramboides* was remarkable in that it claimed the first identification of a purely oligosaccharide-based TH-inducing substance in nature that lacked a significant protein component [1]. Nuclear magnetic resonance spectroscopy (NMR), sugar composition analysis, enzymatic hydrolysis reactions and membrane filtration led to the proposition of a previously unknown xylomannan structure comprised of alternating xylo- and mannopyranosyl units, both with the β-D-(1→4)-configuration (Fig. 1) [1]. Support for this structure was rapidly provided in the form of short synthetic xylomannans with the proposed linkages, whose NMR data were in good but not perfect agreement with those of the natural isolate, with the imperfect matches attributed to the much shorter length of the synthetic glycans and their high proportion of terminal sugars. The absence of TH in the single tetrasaccharide tested was similarly attributed to the inadequate length of the synthetic construct [2–4].

In a tour-de-force of synthetic organic chemistry published recently in *National Science Review* [5], Yu and co-workers have now applied their powerful gold-catalyzed glycosylation methodology to the preparation of multiple oligomeric xylomannans and tested them for TH of ice crystals. To achieve the synthesis of an eicosasaccharide (**1**) corresponding to the proposed structure of the reported antifreeze substance, they adapted the earlier workers’ strategy of preparing a core disaccharide capable of serving both as an acceptor and a donor in their chemistry and dubbed it iterative exponential glycan growth (IEGG). Closely related IEGG strategies were then deployed to assemble a series of alternative xylomannans, replacing D-mannose by its L-enantiomer, the D-mannopyranosyl-(1→4)-D-xylopyranosyl core by its (1→3) linked counterpart and critically preparing multiple oligomers comprised of alternating β-(1→4)-D-mannan and β-(1→4)-D-xylan segments. The NMR data of the eicosasaccharide (**1**) and the octasaccharide assembled by the earlier workers matched and showed the same minor deviations from the natural isolate, dispelling the notion that these arose from a high proportion of terminal units and ruling out the original structure. The literature NMR data of the natural isolate on the other hand correlated well with that of the synthetic block xylomannan structure (**2**), very strongly suggesting it to be the correct structure (Fig. 1). None of the synthetic xylomannans, including the largest block structure (**2**) with an impressive molecular weight of 4.9 kDa, showed TH in the melting of ice crystals thereby rebutting the claim of a protein-free antifreeze oligosaccharide.

The already significant number of natural product structures corrected by total chemical synthesis now extends to the Alaskan *U. ceramboides* beetle xylomannan thanks to the impressive power of modern oligosaccharide synthesis. True protein-free antifreeze glycans remain to be discovered.

***Conflict of Interest Statement***. None declared.
